# Complications of injections in conservative treatment of degenerative spine disease: a prospective unicentric study

**DOI:** 10.1186/s12891-022-05970-x

**Published:** 2022-11-22

**Authors:** Anna Voelker, Markus Pirlich, Christoph-Eckhard Heyde

**Affiliations:** 1 Department for Orthopedics, Trauma and Plastic Surgery, University Hospital Leipzig, Liebigstrasse 20, 04103 Leipzig, Germany; 2 Clinic of Otolaryngology, Head and Neck Surgery, University Hospital Leipzig, Liebigstrasse 20, 04103 Leipzig, Germany

**Keywords:** Spinal injections, Degenerative spine disease, Complications

## Abstract

**Background:**

Spinal injection has been an accepted part of conservative therapy for degenerative diseases. The drugs used can cause side effects and severe complications. The aim of this study was to determine the occurrence of general side effects (GSE) and complications when performing consecutive different types of spinal injections and to evaluate pain reduction.

**Methods:**

Prospective data evaluation of patients with degenerative spine disease at hospital admission, discharge, and six and 12 weeks after discharge. All patients received a specific injection protocol depending on their symptoms and radiological findings. The injections performed were dorsal sacroiliac joint injections, perineural injections, epidural interlaminar and epidural periradicular injections, and facet joint injections. Potential complications were categorized and recorded as GSE and complications. In addition, the Numerical Analog Scale (NAS) for pain, the Oswestry Disability Index (ODI) were evaluated.

**Results:**

Forty-eight patients were enrolled. There were 282 spinal injections performed. A total of 131 common treatment-related events were recorded. Depending on the type of injection, transient pain at the injection site (32.4–73.5%), radiating pain (9.4–34.7%), and nerve root irritation (2–18.4%) were the most common. One complication with postpuncture syndrome occurred with epidural-interlaminar injection. No persistent neurologic deficits occurred. The highest rate of GSE was observed with periradicular injections (relative frequency (RF) = 0.8), followed by epidural-interlaminar injections (RF = 0.65), least frequently with FJ injections (RF = 0.32). From the time of admission to discharge, NAS scores were significantly decreased and ODI score significantly improved at discharge (*p* < 0.001), but relapse occurred at the 12-week follow-up.

**Conclusions:**

Various consecutive spinal injections for conservative treatment of degenerative spine diseases are safe and lead to a decrease in pain and improvement in quality of life. GSE are common, but not persistent. Although complications are rare, they can have serious consequences for the patient.

## Background

Injection of the spine has been an integral part of conservative therapy for degenerative diseases for several years [1]. This may include periradicular injection, facet joint (FJ), and sacroiliac joint (SIJ) injections, and epidural interlaminar and periradicular injections chosen in relation to the underlying spinal pathology. In addition to iatrogenic complications caused by spinal injections, such as injuries to vessels, internal organs, or nerves, the drugs used can cause side effects [2]. Generally, local anesthetics are used for analgesia, and corticosteroids are also used in some types of injections. Local anesthetics induce analgesia by inhibiting the sodium channels and signal transduction. The possible side effects of local anesthetics include central nervous system toxicity, cardiovascular toxicity, and allergic reactions. Sympathetic blockade by injection of local anesthetics can also cause a cardiovascular response [3, 4]. Corticosteroids are used to reduce inflammation by inhibiting phospholipase-2, thus affecting the synthesis of prostaglandins and leukotrienes [5]. Over time, soluble corticosteroids have been replaced by non-soluble corticosteroids in the use of injections [6]. Insoluble suspensions have a retarding effect, but the effect is longer because the active ingredients are released gradually [7]. Minor side effects include transient erythema, facial warmth, and facial flushing [8] as well as increased blood glucose levels in diabetes. The use of epidural cortisone injections, particularly when used frequently, may result in a decrease in bone density and an increase in markers of bone remodeling in postmenopausal women. With a decrease in bone density, the risk of fracture is potentially increased in this group of patients [9, 10].

Due to crystalline suspension, ischemic neurological injuries may occur in the worst case [11]. Allergies or local infections with abscess may also occur. Serious complications (transient or persistent) such as spinal cord infarction, cerebellar infarction, cortical blindness, epidural hematoma, paraplegia, and quadriplegia are described in the literature in case reports, mainly after transforaminal and interlaminar cervical, lumbar, and thoracic injections [12]. The current literature has evaluated side effects of spinal injections only specific to individual types of injections and the drugs used.

This prospective study aimed to determine the overall risk for the occurrence of general and specific complications when performing consecutive different types of spinal injections in the treatment of degenerative spinal disease. The primary study objective was to record general side effects and specific complications due to spinal infiltrations. The secondary study objective was to record pain reduction and change in quality of life due to treatment.

## Methods

### Patient population

**Fig. 1:**
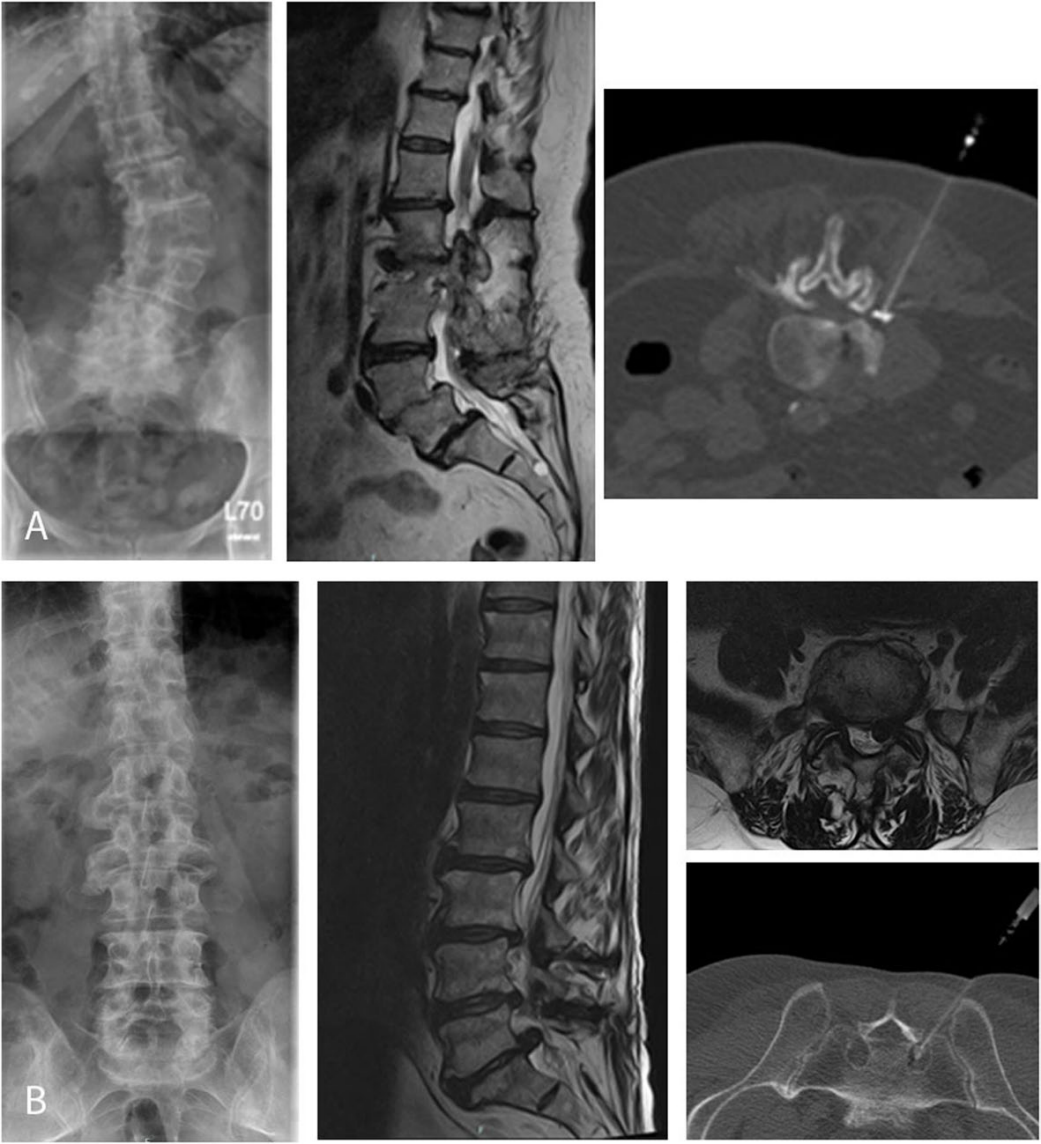
**A** X-ray and sagittal T2-weighted magnetic resonance imaging of degenerated lumbar spine disease with scoliosis and spondylolisthesis (75 years old woman) and ct-guided periradicular injection with contrast of the L3 nerve root on the right side. **B** X-ray and sagittal T2-weighted magnetic resonance imaging of lumbar spine with disc herniation L5/S1 and S1 nerve root stenosis on the left side (59 years old man) and ct-guided periradicular injection with contrast of the S1 nerve root

Hospitalized patients with exacerbation of acute and chronic pain associated with degenerative spine disease who could not be adequately treated as outpatients were enrolled in this prospective study at a single spinal center. The treatment plan for the planned injections was determined by an experienced spine surgeon. Generally, no injection technique type was performed twice on the same patient. All patients were treated per day with a single type of injection in the area of the lumbar spine. The total number of injections that were performed was dependent on the patient's symptoms and image findings. The injections were performed by different orthopedic physicians after special training of injection techniques and under the supervision of a spine surgeon. The computed tomography-assisted periradicular injections were performed by a neuroradiologist (Fig. 1). All facet joint injections and transforaminal epidural injections were done under image-guided control. Injections of the SIJ and epidural injections were performed using anatomic landmarks. Clinical follow-up was performed at six and 12 weeks after admission.

After inpatient injection therapy, the dose of analgesics was not increased, but was decreased whenever possible if less pain was present. Physical therapy was recommended depending on persistent symptoms, but not prescribed.

Inclusion criteria were age > 18 years, subacute or chronic pain due to degenerative spine disorders, and no indication for urgent surgical therapy. Exclusion criteria were age < 18 years, pregnancy, and acute spinal diseases requiring specific treatment such as neurological deficits, inflammation, fractures, or tumors of the spine. In addition, patients with open wounds, intolerance to local anesthetics or corticosteroids and neurologic diseases were excluded, as well as patients with severe cardiovascular disease and coagulation disorders or taking anticoagulant medications.

Written informed consent was obtained from all the patients. A positive vote from the Ethics Committee of the Medical Faculty of the University of Leipzig was available (177–2009-17,082,009), and the study was conducted in accordance with the Declaration of Helsinki.

### Analyzed parameters

The following injections were performed depending on the symptoms and radiological findings of the included patients: dorsal SIJ injection, perineural injections including transforaminal epidural injections, epidural interlaminar and epidural periradicular injections, and FJ injections. Possible complications were classified into general side effects and specific complications, and were recorded and evaluated for each individual injection [1, 13–15].

The patient-specific parameters, such as age, sex, and weight, were analyzed. Patient complaints were classified as acute (less than six-week duration) or chronic (> 12 weeks). Clinical results were evaluated during inpatient admission, discharge, after six weeks, and after 12 weeks after admission.

In addition, the data on the following parameters were obtained and evaluated during hospital admission, discharge, and at six and 12 weeks after discharge: the Numeric Analogue Scale (NAS) for pain, Oswestry Disability Index (ODI), and laboratory values for inflammation (C-reactive protein [CRP] and leukocytes [LC]) to monitor systemic infection.

### Statistical analysis

Graphs and analyses were generated using Microsoft Office 2019 (Microsoft Corporation, Redmond, WA, USA) and GraphPad Prism Software 9 (GraphPad Software, La Jolla, CA, USA). Data are presented as mean ± standard deviation (SD). The significance of the mean values was tested using unpaired t-test when the values were normally distributed and Mann–Whitney U-test when the values were not normally distributed. Fisher’s exact test was used when the sample size was small.

## Results

### Patient characteristics

Forty-eight consecutive patients were enrolled in this prospective study. The mean age of the patients was 58.38 ± 12.5 years (range 30–84 years). The female-to-male ratio was 1:1. The mean hospitalization stay was 6.25 ± 1.44 days (range 4–11 days). The overall mean number of injections for each patient was 5.88 ± 1.1 (range 4–9). In total, 282 spinal injections were performed and analyzed.

The reasons for inpatient admission to spinal infiltration therapy varied. Thirty-four patients (70.83%) had chronic lumbar spine syndrome, five (10.41%) had lumbar disc herniation or spinal stenosis, four (8.33%) had chronic lumbar spine syndrome after previous surgery, three (6.25%) had chronic cervical spine complaints, and two had other underlying conditions (Scheuermann's disease and ankylosing spondylitis).

Most patients suffered from back pain with a limited range of motion (93.8%), followed by sensory deficits (68.8%), ischiatic pain (50%), positive leg-raise test (35.7%), and chronic motor deficits (10%). Analysis of clinical symptoms over 12 weeks showed improvement in all parameters after injection therapy. Most improvements were observed between hospital admission and discharge. Sensory deficits were reduced by approximately 43.3%, ischiatic pain by 39.4%, limitation of motion due to pain by approximately 30%, positive leg-raise test by approximately 22.9%, and motor deficits by approximately 14.4%. After 12 weeks, the clinical symptoms recurred. Detailed values are presented in Table 1.

**Table 1:** Change in clinical symptoms in the study population

	Admission n (%)	Discharge n (%)	6-week follow-up n (%)	12-week follow-up n (%)
Limited range of motion	45 (93.8)	31 (63.8)	40 (83.7)	40 (82.5)
Ischiatic pain	24 (50)	6 (10.6)	10 (18.6)	14 (20)
Positive leg raise test	17 (35.7)	6 (12.8)	10 (20.9)	12 (25)
Sensory deficits	33 (68.8)	12 (25.5)	20 (41.9)	22 (47.5)
Motor deficits	12 (25)	5 (10.6)	9 (18.6)	10 (20)

### Injections

In total, 282 spinal injections were administered. In descending order of frequency, 114 fluoroscopy-assisted infiltrations of the FJs (bilateral), 50 injections of the SIJs, 49 periradicular injections (computed tomography [CT] -assisted, fluoroscopically assisted), 37 epidural-interlaminar injections, and 32 epidural-perineural injections were administered. A detailed list of the medications used is shown in Table 2.

**Table 2:** Medication use based on the injection type

	Periradicular, n (%)	FJ (bilateral), n (%)	SIJ (bilateral), n (%)	Epidural interlaminar n (%)	Epidural perineural, n (%)
n	49	114	50	37	32
Anesthetic	17 (34.7)	54 (47.8)	28 (56)	11 (29.6)	13 (40.9)
Anesthetic + steroid	16 (32.7)	60 (52.2)	22 (44)	26 (70.4)	19 (59.1)
Anesthetic + steroid + contrast medium	16 (32.7)	0	0	0	0

*FJ* facet joint, *SIJ* sacroiliac joint

### Analyzed follow-up parameters on pain, quality of life, and infection

The performed spinal injections resulted in a statistically significant reduction of NAS values from the date of admission (NAS = 6.83 ± 1.53) to discharge (NAS = 3.64 ± 1.86; *p* < 0.001). At the 12-week follow-up, there was a rebound in NAS values, but the values were lower than those at admission (6.83 vs. 5.42). Analysis of the ODI values showed similar results. Between admission and discharge, there was a significant improvement in the ODI score (48.04% vs. 33.57%, *p* < 0.001). However, at 12 weeks of follow-up, the ODI increased to 44%. Analysis of inflammatory values (CRP and LC) showed no significant changes in the collected values at 12 weeks of follow-up (Table 3).

**Table 3:** Change in parameters on pain, quality of life, and infection across 12 weeks

	Admission n	Discharge n	6-week follow-up n	12-week follow-up n	*p*-value
NAS	6.83	3.64	4.77	5.42	< 0.001
ODI	48.04	33.57	39.21	44	< 0.001
CRP	2.43	2.4	1.7	2.7	*p* > 0.05
LC	7.3	8.57	7.26	7.29	*p* > 0.05

*NAS* numerical analogue scale, *ODI *Oswestry Disability Index, *CRP *C-reactive protein, *LC *leucocytes

### Side effects and complications after spinal injections

General side effects were observed for all injection types. In total, 131 common treatment-related events were recorded. The largest number of events had a symptom duration of < 10 h (*n* = 91, 65.9%). Symptoms lasting > 10 h occurred in 43 (31.2%) patients, and on the day of discharge, symptoms due to injection were still present in four patients (2.9%). At the six- and 12-week follow-up, no treatment-related general side effects were observed.

Depending on the injection technique used, reported side effects occurred at varying frequencies. Transient local pain (< 10 h) at the injection site occurred in all infiltration types (32.4%–73.5%). Similarly, radiating pain (9.4%–34.7%) and sensory deficits (2%–18.4%) occurred in all groups. Both symptoms occurred mainly with injections into the nerve root region. Temporary headache was observed most frequently in the epidural-interlaminar group (13.5%) and least frequently in the FJ-injection group (1.8%). Mild circulatory dysregulation occurred in all groups (2.7%–4.1%), except in the FJ group. No severe systemic circulatory disturbances, systemic or local infections, or persistent neurologic disturbances were observed after the spinal injections. Intrathecal application with post-puncture syndrome occurred during epidural-interlaminar injection as a specific complication. Other specific complications, such as local and systemic infections, myelopathy, stellate blockade, pneumothorax, paraplegia, and renal puncture, did not occur. A detailed list of all observed side effects and specific complications is presented in Table 4.

**Table 4:** General side effects after different spinal injections

**General side effects**	Periradicular injection (*n* = 49), n (%)	FJ injection (*n* = 114), n (%)	SIJ injection (*n* = 50), n (%)	Epidural perineural injection (*n* = 32), n (%)	Epidural interlaminar injection (*n* = 37), n (%)
Headache	1 (2)	2 (1.8)	1 (2)	3 (9.4)	5 (13.5)
Nausea	0	0	3 (6)	0	1 (2.7)
Vomiting	0	0	0	0	1 (2.7)
Mild circulatory regulation disturbance	2 (4.1)	0	3 (6)	1 (3.1)	1 (2.7)
Severe circulatory regulation disturbance	0	0	0	0	1 (2.7)
Allergic reaction	1 (2)	0	2 (4)	0	0
Abscess	0	0	0	0	0
Oedema	1 (2)	0	1 (2)	0	0
Transient sensory deficit	9 (18.4)	5 (4.4)	1 (2)	4 (12.5)	3 (8.1)
Pain on site of injection (< 10 h)	36 (73.5)	64 (56.1)	27 (54)	15 (46.9)	12 (32.4)
Pain on site of injection (> 10 h)	2 (4.1)	5 (4.4)	1 (2)	0	1 (2.7)
Radiating pain	17 (34.7)	19 (16.7)	12 (24)	3 (9.4)	6 (16.2)
Transient bladder dysfunction	5 (10.2)	3 (2.6)	0	0	3 (8.1)
Transient motor deficit	0	2 (1.8)	1 (2)	2 (6.3)	0
Myalgia	1 (2)	0	0	0	0
Hematoma	0	1 (0.9)	0	0	0
Meningism	0	0	0	0	1 (2.7)
Others	0	0	0	0	1 (2.7)
**Specific complications**					
Post-puncture syndrome	0	0	0	0	1 (2.7)

*FJ* facet joint, *SIJ* sacroiliac joint

Analysis of the relative frequency of general side effects or specific complications per spinal injection type showed the highest rate of general side effects with periradicular injections (relative frequency [RF] = 0.8), followed by epidural interlaminar injections (RF = 0.65). General side effects were rarest with FJ injections (RF = 0,32), Table 5.

**Table 5:** Relative frequency of general side effects and specific complications

**Type of injection**	Number of injection (n)	GSE (n)	GSE: relative frequency (RF)	SC (n)	SC: relative frequency (RF)	*p*-value
Periradicular injection	49	39	0.8	0	0	-
FJ injection	114	37	0.32	0	0	-
SIJ injection	50	25	0.5	0	0	-
Epidural perineural injection	32	13	0.41	0	0	-
Epidural interlaminar injection	37	24	0.65	1	0.03	< 0.001

*GSE *general side effects, *SC *specific complications, *FJ *facet joint, *SIJ *sacroiliac joint

Furthermore, we compared the number of general site effects in periradicular injection with the other injection techniques. We found that periarticular injection resulted in a significantly higher incidence of GSE than facet joint injection (*p* < 0.001), SIJ injection (*p* = 0.0031), and epidural periradicular injection (*p* = 0.0007) (Table 6).

**Table 6:** Incidence of general side effects of each injection technique compared with observed general side effects of periradicular injection

	GSE	*p*-value
P_I (*n* = 49): FG_I (*n* = 114)	39:37	< 0.001
P_I (*n* = 49): SIJ_I (*n* = 50)	39:25	0.0031
P_I (*n* = 49): EI_I (*n* = 37)	39:24	0.1461
P_I (*n* = 49): EP_I (*n* = 32)	39:13	0.0007

*GSE *general side effects, *P*_*I *periradicular injection, *FJ*_*I *facet joint injection, *SIJ*_*I* sacroiliac joint injection, *EI*_*I* epidural interlaminar injection, *EP*_*I* epidural perineural injection

## Discussion

The aim of the study was to determine prospectively the general rate of side effects as well as specific complications in the context of inpatient injection therapy with different types of injections in the treatment of degenerative lumbar spine disorders.

The results of this study show that the use of injections, based on the specific complaints in accordance with radiological findings, improved clinical symptoms, NAS, and ODI in the short term. This is consistent with the literature on conservative therapy for degenerative spinal disorders [16–19].

Serious complications have been described in literature. These include infections, hematomas, intravascular injections, nerve injury, dura puncture, air embolisms, and their associated clinical signs [14].

Side effects or minor complications occurred with all injection types in our study population. The range of relative frequency of general side effect was 0.32 to 0.8 overall. Various general side effects were observed with the periradicular injection technique. These included pain at the infiltration site, radiating pain, and temporary sensory deficits. These complaints are attributed to the application of the drugs directly to the corresponding nerve root or to local tissue trauma caused by needle placement or mild hematoma beside others.

In a review of complications after transforaminal epidural injection, Change et al. reported a minor complication rate of 2.4%–9% [20]. This finding is inconsistent with the results of our study. Short-term pain at the injection site was present in 73.5% of the patients. This difference could be because the included studies did not explicitly distinguish between short- and long-lasting pain. In addition, only studies that included steroid injections were analyzed. This explains why transient sensory deficits occurred only in our patients, as anesthetic and steroid were used for the injections.

Over the years, image-guided transforaminal injection using either CT, MRI, or X-ray has become standard. In a study between ct-assisted and anatomical landmark-guided injection, Demel et al. showed that ct-assisted periradicular injection resulted in a higher accuracy of needle position with better pain reduction. Major complications did not appear in both groups [21]. Kamp et al. could not find any complications in a study of ct-assisted vs fluoroscopy-assisted transforaminal epidural steroid injection for lumbar radiculopathy in 116 patients [22]. Goodmann et al. recommend the use of contrast when performing image-guided transforaminal epidural injections to avoid intraarterial penetration and possible associated application of particulate steroids into the artery of adamkiewicz [14]. In our study group, contrast was used only in 33% of all periradicular injections. Causes for this could be an allergy to contrast agent or limited renal function. Severe neurological complications after injection did not occur in this group. However, it should be discussed whether contrast medium should generally be used for transforaminal epidural injection in the absence of contraindications.

The side effects of transforaminal epidural injection with steroids include vascular penetration, non-positional headache, back pain, worsening leg pain, facial flushing, hypertension, transient nerve root irritation, and vasovagal reactions [15]. Our results are consistent with the results in this study. The patients with periradicular injection in our study also complaint of headache, worsening leg pain, milde circulatory regulation disturbance and transient nerve root irritation.

Dura puncture with subsequent injection into the subdural and subarachnoid spaces, resulting in complications such as cauda equina and conus medullaris syndromes, persistent paresthesia, infections, ascending weakness/loss of sensation, apnea, and unconsciousness, did not occur in our patients [13].

SIJ and FJ injections are low-side-effect procedures. This is consistent with our data and shows up with the lowest RF of 0.32 compared to the other injections. Our patients mainly experienced short-term pain at the injection site and radiating pain. In a systematic review of FJ interventions, Boswell et al. showed only minor complications with the use of radiofrequency procedures in the FJs, but not in injections [16]. Feared complications of facet joint injections are accidental puncture of the dura, spinal anesthesia, injury to adjacent organs or injury to neural structures. Reliable data regarding the incidence of these complications could not be found even with Boswell's review about effectiveness and complications of facet joint interventions [1]. Image-guided injection of the facet joints, as also performed in our study, can minimize needle misplacement and associated complications. In addition, image-guided application achieves control of the target region. Infections described as isolated in the literature did not occur in our study population [23, 24].

Epidural perineural and interlaminar injections had common side effects in our study. Local pain at the injection site was also the leading cause of pain. Headache was the most common symptom in both groups. In addition, disturbances in circulatory regulation have occurred. This is due to the effect of the steroids used and has been described in the literature [25].

Temporary bladder dysfunction was also observed in few patients in the epidural interlaminar and periradicular injection groups. This can be explained by the fact that the administration of local anesthetics in the region of the lumbar and sacral nerve roots is associated with a higher incidence of urinary retention [14].

In a systematic review, Vorobeychik et al. categorized the complications and side effects of non-image-guided lumbar interlaminar steroid injections into different categories. For this purpose, four groups were formed: technique, infection, steroid-use, and allergy. It is noted in the study that the described complications are only found in case reports in the literature. They concluded that only individually reported cases indicated high safety of the procedure [26]. Dura puncture may occur during epidural interlaminar and periradicular injections. The reason for this is the placement of the needle in the epidural space. Blockages in neural structures may occur when anesthetic is applied. Transient sensory or motor deficits usually occur. Isolated cases of respiratory depression have been described when high doses of anesthetics are applied [27, 28].

Headache may also occur due to dural puncture. This could hypothetically be the reason for the high number of headache cases in the patient group [29]. However, it should be noted that only one patient had typical post-puncture syndrome as a specific complication of epidural interlaminar injection. Post-lumbar headache is reported in the literature in the range of 3.5%–33% [30]. Accidental puncture of the dura during injection can be avoided by accurate needle placement when using the epidural injection technique.

Intravascular application of anesthetics or steroids was not explicitly investigated in our patient group. Only few of the periradicular injections were performed with the application of contrast medium. The distribution of the contrast agent at injection was not explicitly studied.

The individual cases that are described with serious complications due to the intravascular application of steroids, especially particulate corticosteroids, show that careful needle placement is also necessary. Although digital subtraction angiography is a standard technique for detecting intravascular application of transforaminal epidural injections, it cannot prevent serious neurological complications in individual cases [31].

Hong et al. showed that the diameter of the needle used for infiltration has a significant influence on the incidence of intravascular application. Using digital subtraction angiography, a lower incidence of intravascular application was demonstrated with the Whitacre needle (5.4%) than with the Quincke needle (16%).

However, this study did not differentiate between venous and arterial applications. A differentiation would be interesting from the authors' point of view since the intravascular application of insoluble steroids can cause paraplegia or ischemia in individual cases [32].

The strengths of the study are its prospective study design, with a follow-up of up to three months after intervention. In addition, different types of consecutive injections were administered to patients within a specific duration. Another advantage is that we distinguished between general side effects and complications and recorded them explicitly after each infiltration. The disadvantages are the small number of patients and the small number of different types of infiltrations. We also did not evaluate the side effects or complications in terms of the drugs that were used. Further studies should be performed with a larger patient population, a detailed analysis of the applied drugs in terms of side effects, and a radiological evaluation of the distribution of contrast medium (if applied).

Our study shows that even multiple consecutive infiltrations in the treatment of degenerative diseases of the spine has no accumulation of complications. Despite the frequent occurrence of side effects after injections, there is a significant reduction in pain and improvement in quality of life because of treatment in short-term follow-up. However, in the long-term study, the values obtained converge again.

## Conclusions

In summary, spinal injections for conservative treatment of degenerative diseases are safe and lead to a decrease in pain and improvement in quality of life. However, for each medical procedure, patients must be informed regarding the possible serious complications and the common occurrence of general side effects. Careful performance of injection techniques with accurate needle placement is essential for decrease in pain and complication rates.

## Data Availability

All relevant data generated or analyzed during the current study are presented in the paper. The detailed datasets used during the current study are available from the corresponding author upon reasonable request.

## Abbreviations

GSE: General side effects
NAS: Numerical Analog Scale
ODI: Oswestry Disability Index
RF: Relative frequency
FJ: Facet joint
SIJ: Sacroiliac joint injections
CRP: C-reactive protein
LC: Leukocytes
SD: Standard deviation
